# Diversity and Ecology of Late Glacial Diatoms of the Eastern Baltic Region

**DOI:** 10.3390/biology14091226

**Published:** 2025-09-09

**Authors:** Anna Rudinskaya, Olga Druzhinina

**Affiliations:** 1 Institute of Geography, Russian Academy of Sciences, 119017 Moscow, Russia; 2 Faculty of Geography, Herzen University, 191186 Saint-Petersburg, Russia; olga.alex.druzhinina@gmail.com

**Keywords:** diatom analysis, paleolimnology, paleogeographic reconstructions, Late Glacial, Sambian Peninsula, eastern Baltic

## Abstract

The sediments of shallow basins formed on along the coast of the Baltic Ice Lake in the Late Glacial are a valuable source of information for reconstructing the paleoenvironment. A detailed diatom analysis, along with chronological and lithological studies, was performed for the sediments of one of these paleoreservoirs, uncovered in the Kulikovo section (Sambian Peninsula, Kaliningrad Region of the Russian Federation) and dated to 14,000–12,500 calBP. As a result of this study, the essential diversity of Late Glacial diatoms was revealed and 204 species were found. Among them, benthic and epiphytic species belonging to the group of oligohalobous indifferents predominate. The most typical species are *Pseudostaurosira brevistriata*, *Staurosirella ovata*, *Gyrosigma attenuatum*, *G. acuminatum*, *Amphora affinis, Epithemia adnata* and *Cymbopleura inaequalis*. A comparison with available regional data showed that the mass development of pioneer epiphytic diatoms of the Fragilariaceae in the Allerød is common to most of the paleoaquatic ecosystems. A local feature of the Kulikovo paleoreservoir, along with a wide range of ecological diatom groups, is the episode of massive development of *Gyrosigma* spp., benthic diatoms capable of living in streaming water, in the first half of the Allerød. The Kulikovo diatom assemblages reacted sensitively, not only to the main environmental shifts of the Allerød and Younger Dryas, but also to smaller-scale climatic events, such as Herzensee Oscillation (GI-1b) and changes in hydrological regimes (water level fluctuations, flow rate) caused by local environmental transformations.

## Simple Summary

Here, we present a manuscript devoted to the results of the Kulikovo sediment section (southeastern Baltic) diatom study. We provide a reconstruction of local aquatic environments of the Bølling–Allerød transition and Younger Dryas (14,000–12,500 calBP). The uniquely preserved deposits of this section, representing one of the post-glacial basins, made it possible to reconstruct in high-resolution the changes in aquatic organisms of the Late Glacial. In most of the regional sedimentary archives, the number of diatom species in the Late Glacial sediments does not exceed several dozen, while we identify 204 taxa due to the high resolution of our sampling (every 1–3 cm). This research also showed an unexpected diversity of diatoms for the Late Glacial. Thus, in this article, we present the most detailed description of the Late Glacial diatom assemblages of the Baltic region that currently exists. We believe that it will be interesting to a broader international audience studying aquatic biota and its responses to paleoclimate changes of the Late Glacial, as well as the history of the Baltic Sea, especially its initial stages.

## 1. Introduction

The southeastern Baltic was covered by glaciers during the Last Glacial Maximum [1]. The natural environment of this region has undergone major changes since the beginning of deglaciation 20,000–19,000 calBP [2,3,4,5], since it was significantly influenced by climatic fluctuations of the Bølling–Allerød and the Younger Dryas. Diatoms, as one of the biotic components of aquatic systems sensitive to natural climatic changes, are a valuable source of information to trace the environmental transformations of the past.

In general, information on the Late Glacial (especially for the Bølling–Allerød) diatom assemblages in the eastern Baltic, as well as in the broader region of the northwestern part of the European plain, cannot be considered sufficient due to the small number of species found or often poor preservation of the valves [5,6,7,8,9,10]. Diatoms attributed to Bølling–Allerød sediments have mainly been found on the territory of Lithuania in large ancient lakes and in the sediments of the Baltic Ice Lake (BIL). M.Kabailiné [6,7,8] created a regional stratigraphic scheme of the Late Glacial in Lithuania and identified three diatom zones. In studies of the last two decades on the territory of Lithuania [11,12,13,14,15], Poland and the Kaliningrad region [5,16,17,18,19], new data on Late Glacial diatoms of this area have been collected.

The oldest diatom zone (DZ1) in sediments of continental lakes corresponds to deposits of the Oldest Dryas, Bølling and Older Dryas. Usually, the valves are not very abundant—their concentrations are less than 1000 valves per cm^3^. According to [8], the diatom flora of this chronozone is represented mainly by valves of freshwater planktonic (*Cyclotella*, *Aulacoseira*) and benthic species (*Amphora pediculus*), which prefer cold and moderate temperature conditions in oligotrophic and mesotrophic water bodies. At the same time, eutrophic freshwater cosmopolitan epiphytic diatoms, such as *Pseudostaurosira brevistriata*, *Staurosirella pinnata*, and *Sraurosira construens* [13,15,16], have been found in the sediments of this period.

In the Allerød chronozone (DZ2), diatoms are represented mainly by mesotrophic-eutrophic freshwater epiphytic species (*Pseudostaurosira brevistriata*, *Staurosirella pinnata*, *Staurosira construens*, etc.) and benthic diatoms. Several species of diatoms have been found in the Allerød sediments. (According to [6], up to 90 species of diatoms were identified in some paleoreservoirs.) At the same time, the Allerød diatoms in the studied sections show some differences. For example, in Lake Ginkūnai sediments [13], the content of planktonic diatoms increases to 10% from 13,700 calBP, moist habitats species indicating a slight increase in the depth of the paleoreservoir and flooding shores, and warm-water species of the genus *Navicula* spp. appear. In the first half of the Allerød, the pioneer epiphytic species belonging to the genera *Fragilaria* and benthic species (*Navicula* spp., *Amphora pediculus*) predominated in the sediments of the lakes Trzechowskie [18], Petrašiūnai [14] and Kašučiai [15]. Numerous Allerød diatoms assemblages of the bottom sediments of Lake Petrašiūnai were boreal species and preferred warm temperature conditions. In the second part of Allerød (from 13,200 to 12,900 calBP), there was an increase in the content of planktonic species in the bottom sediments of lakes Trzechowskie [18], Petrašiūnai [14], Kašučiai and Pamerkai [15]. That means the depth of these paleoreservoirs slightly increased. Planktonic diatoms predominated (up to 70% of all valves) in the bottom sediments of Lake Varenis due to cold oligotrophic conditions [12]. In the Aleika section [19], pioneer epiphytic species of Fragilariaceae predominated from 13,100 to 12,900 calBP, and at about 12,800 calBP, the content of benthic *Gyrosigma* spp. increased.

Common features are also highlighted for Younger Dryas diatom assemblages. Planktonic oligotrophic diatoms (*Cyclotella* spp.) predominated in this period, and benthic *Gyrosigma*, *Cymatopleura* spp. and epiphytic *Fragilaria* spp. were widely represented. Younger Dryas diatom assemblages consist of several dozen species—for example, more than 40 species in the Pamerkai outcrop [15]. Planktonic and cold-water diatoms prevail, which shows increasing paleoreservoir depth and decreasing water temperature [7,13].

For the Baltic Sea deposits, BIL Younger Dryas diatoms assemblages [6] consist mostly of freshwater species (up to 70–80%), although there some halophilous and marine species. The most widespread species were *Ellerbeckia arenaria*, *Staurosirella martyi*, *Pseudostairosira brevistriata*, *Staurosirella pinnata*, *Diploneis burgitensis*, *Gyrosigma attenuatum*, *Navicula oblonga*, *Cavinula scutelloides*, *Cymbopleura inaequalis*, *Amphora mexicana var. major*, *Mastogloia elliptica*, *Mastogloia danseyi*, and *Iconella hibernica*.

**Figure 1:**
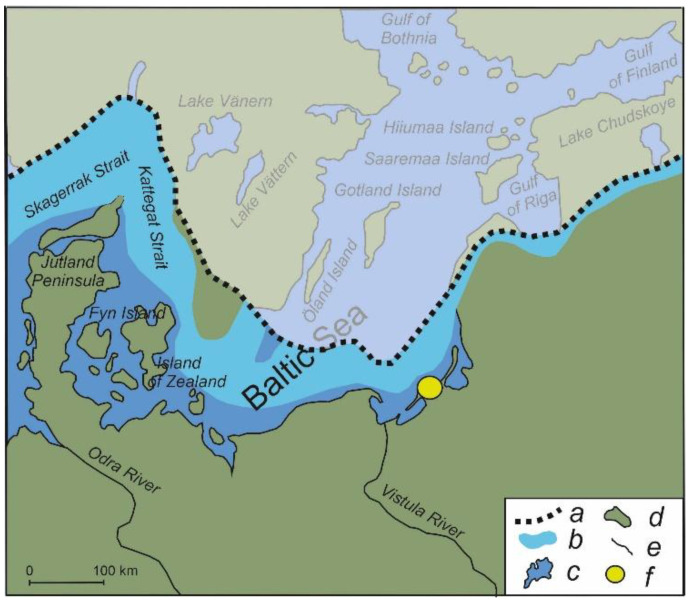
Location of the Kulikovo sedimentary archive. Legend: a—southern border of the Scandinavian glacier around 14,500 calBP [21]; b—Baltic Ice Lake about 14,500 calBP [21], c—modern Baltic Sea, d—modern land, e—rivers, f—Kulikovo section.

Thus, the dynamics of diatom assemblage composition of the reviewed continental paleoreservoirs in the eastern Baltic demonstrate some asynchrony. This indicates different times and degrees of response of aquatic ecosystems to changes in natural conditions. In this context, the paleoreservoir Kulikovo (Figure 1), a new sedimentary archive of the southeastern Baltic, is of particular interest. Previous investigations [20] established a sensitive reaction of this paleoreservoir’s biota to short-lasting Late Glacial warmings and coolings, as well as changes in flow rate, depth and other factors, possibly, due to the shallow depth of this paleoreservoir. Therefore, studying the Kulikovo deposits can give detailed information on regional and local trends in environmental dynamics. The aim of this study is to present results of a high-resolution diatom analysis of the Kulikovo section deposits for the interval from 14.0 to 12.5 ka and to complement and refine the existing ideas about the diatom flora of the eastern Baltic, which can provide a better understanding of how aquatic organisms reacted to significant climate and environment change in the Late Glacial.

## 2. Materials and Methods

### 2.1. Geographical Setting

The Kulikovo section is located in the west part of the Kaliningrad region, Russia, in the coastal cliffs of the Sambian Peninsula (54°56′13″ N, 20°21′30″ E). The region is located in the southwestern part of the Baltic syneclise, within the East European platform. The modern relief of this area was formed as a result of the direct activity of the Last Glacial glaciers and limno- and fluvioglacial processes. The Sambian Peninsula is characterized by rather complex forms: natural complexes of hilly, hilly–ridged, moraine and rock relief alternate with flat and slightly undulating moraine and lake–glacial plains. In this part of the Baltic, various types of soils are formed under meadow, marsh and forest vegetation (sparse pine forests, spruce, broadleaf forests, as well as black alder forests)—from swampy lowland humus-peat to brown forest. The climate is characterized by excessive humidification, mild winters with an average January temperature of 4.5 °C, and mild summers with an average July temperature of +17.5 °C [22].

### 2.2. Geochronology and Lithology

To determine the absolute age of the sediments for the entire section (192–0 cm), a series of five radiocarbon dates was obtained (Table 1). The dating material is represented by gyttia and wood remains. The data obtained for ^14^C has been adjusted to ^13^C, the deviation from the agreed standard value of the ^13^C/^12^C ratio was taken into account. The age–depth model was built using the rbacon 3.1.0 program [23]. All radiocarbon dates were calibrated using the IntCal20 Northern Hemisphere Radiocarbon Age Calibration Curve (0–55 cal kBP) [24]. A comprehensive lithological analysis was previously performed to reconstruct the sedimentation conditions [25]. The thickness of the deposits exposed in the Kulikovo section is 2 m. The sediments of undisturbed texture were sampled in metal boxes 7 cm wide and 50 cm long. Next, samples were taken in the laboratory at intervals of 1–3 cm.

### 2.3. Diatom Analysis

Diatom analysis was performed for 67 samples. Slides were prepared according to the standard procedure [26]. From 1 to 2 g of a natural moisture sample was treated with 10% hydrochloric acid and 30% hydrogen peroxide to remove carbonates and organic matter, and then the clay fraction was removed by elutriation (6 times with an interval of 4 h). Next, the density separation of the samples was carried out using heavy liquid with a density of 2.3 g/cm^3^. We added one lycopodium tablet to each sample (Batch 280521 291–13,761 spores in one tablet without taking into account the standard deviation) to calculate the concentration of valves (valves/g of the material). The dry weight of each sample was calculated from the weight moisture content of the sample measured during lithological analyses. The weight concentration of diatoms was calculated according to the following formula [26]:

Concentration, valves/g = (N_L_ × n_d_)/(n_L_ × w_dry_) (1)

where N_L_ is the total number of spores in each *Lycopodium* tablet, n_L_ is the number of *Lycopodium* spores counted in the slide, n_d_ is the number of diatom valves counted in the slide, and w_dry_ is the dry weight of the sample.

In each slide, from 100 to 1200 valves were identified. Identification was performed using a Motic BA 300 electronic binocular at 1000× magnification. Differences in the number of identified valves for each preparation are associated with different saturation of the samples with valves. For the identification of taxa, we used “Key to Diatoms of Russia” [27] and “Bacillariophyceae” [28,29,30,31]. The names of the identified diatom species were verified using the resource https://www.algaebase.org/ (accessed on 30 July 2025) [32].

**Figure 2:**
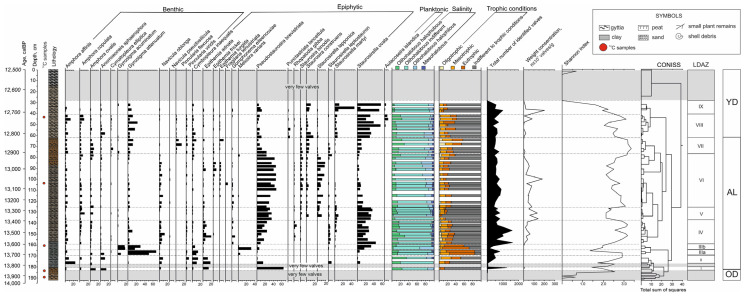
Diatom percentage diagram of selected taxa (their proportion in at least one sample is equal to or exceeds 5% of all identified valves), and correlations between ecological groups of diatoms of the Kulikovo section. The following abbreviations are used in the figure: CONISS – the results of stratigraphically constrained cluster analysis, LDAZ—local diatom assemblage zone, OD—Older Dryas, AL—Allerød, YD—Younger Dryas.

The diatom diagram (Figure 2) was built using the TILIA 2.6.1 software package. It shows selected taxa with a proportion in at least one sample equal to or exceeding 5% of all identified valves. The division into local diatom assemblage zones (LDAZs) was performed based on the results of cluster analysis by CONISS [33]; the Euclidean distance of 10 was chosen for the allocation of clusters. Among the identified species of diatoms, ecological groups were distinguished according to their habitat, according to the Kolbe–Hustedt halobian system, and depending on the trophic status of the water body that is most preferable for the species.

By habitat, the identified diatom species were divided into three types: (i) benthic species living on bottom soil and capable of movement; (ii) epiphytic species forming biofouling from solitary and colonial forms on the bottom, underwater rocks, algae and living organisms; and (iii) planktonic species, passively drifting in the water column [34].

When grouped according to the Kolbe–Hustedt halobian system [35,36], the identified species were divided into two groups: (i) mesohalobous (brackish water) species preferring salinity from 5 to 20‰; and (ii–iv) oligohalobous, i.e., freshwater, species that prefer a salinity of 0–5‰. Among the oligohalobic species, the following groups were distinguished: (ii) halophobous species, which prefer exclusively freshwater habitat conditions; (iii) indifferent species capable of living in conditions of increased mineralization; and (iv) halophilous species that are highly vegetative with high mineralization.

When grouping species depending on the trophic conditions of the reservoir [34], which is most preferable for diatoms of the selected taxa, the following groups were identified: (i) diatoms that prefer oligotrophic water bodies with cold, well-oxygenated, clear water and a low content of dissolved nutrients; (ii) diatoms that prefer eutrophic-type water bodies, well warmed up and characterized by high productivity and a high content of biogenic elements; (iii) diatoms that prefer water bodies of medium trophicity (mesotrophic); and (iv) diatoms indifferent to the trophicity of the habitat.

To assess species diversity, the Shannon diversity index was used, calculated using the following formula [37]:

H = Σp_i_ × log_2_p_i_, (2)

where H is biodiversity, and p_i_ is the specific abundance.

## 3. Results

### 3.1. Geochronology and Lithology

The results of radiocarbon dating are presented in Table 1. The age for each centimeter of the section was calculated using the depth–age model. A sampling interval of 1–3 cm provides a temporary resolution of samples of about 6–30 years. All radiocarbon dates are within the 95% confidence interval range. According to the depth–age model, the sediments comprising the section began to accumulate at 14,038 ± 140 calBP. The uncertainty interval for the lowest part of the section (192–145 cm) is ±130–160 years, and higher along the section it increases to ±170–190 years.

The lithostratigraphic description of the section is presented in Table 2. The lithological classification of types of bottom sediments used is based on the content of organic matter in the sediments [38]. Based on the results of a comprehensive lithological analysis of the Kulikovo section, it was found that the silt fraction prevails at all depths (for details see [24]).

### 3.2. Diatom Analysis

A detailed description of the diatom flora is given in Appendix A—Table A1. The following families are most widely represented: Cymbellaceae (27 species), Naviculaceae (23), Stauroneidaceae (14), Bacillariaceae (13), Gomphonemataceae (13), Pinnulariaceae (13), Staurosiraceae (13), Surirellaceae (13), Achnanthidiaceae (10 видов), Rhopalodiaceae (8), Stephanodiscaceae (7), Fragilariaceae (6), Sellaphoraceae (6), Encyonemataceae (5), Eunotiaceae (5), Catenulaceae (4), Neidiaceae (3), and Ulnariaceae (3). Other families are represented by 1–2 species. The dominant genera are represented by *Gomphonema* (14 species), *Stauroneis* (14), *Pinnularia* (12), *Cymbella* (11), *Navicula* (11), *Cymbopleura* (9), *Caloneis* (7), *Placoneis* (7)*, Surirella* (7), *Epithemia* (6), *Nitzschia* (6), *Staurosira* (6), *Encyonema* (5), *Eunotia* (5), *Sellaphora* (5), *Amphora* (4), *Iconella* (4), *Staurosirella* (4), *Fragilaria* (3), *Hantzschia* Grunow (3), *Neidium* (3), *Planothidium* (3), *Tryblionella* (3), and *Ulnaria* (3).

**Figure 3:**
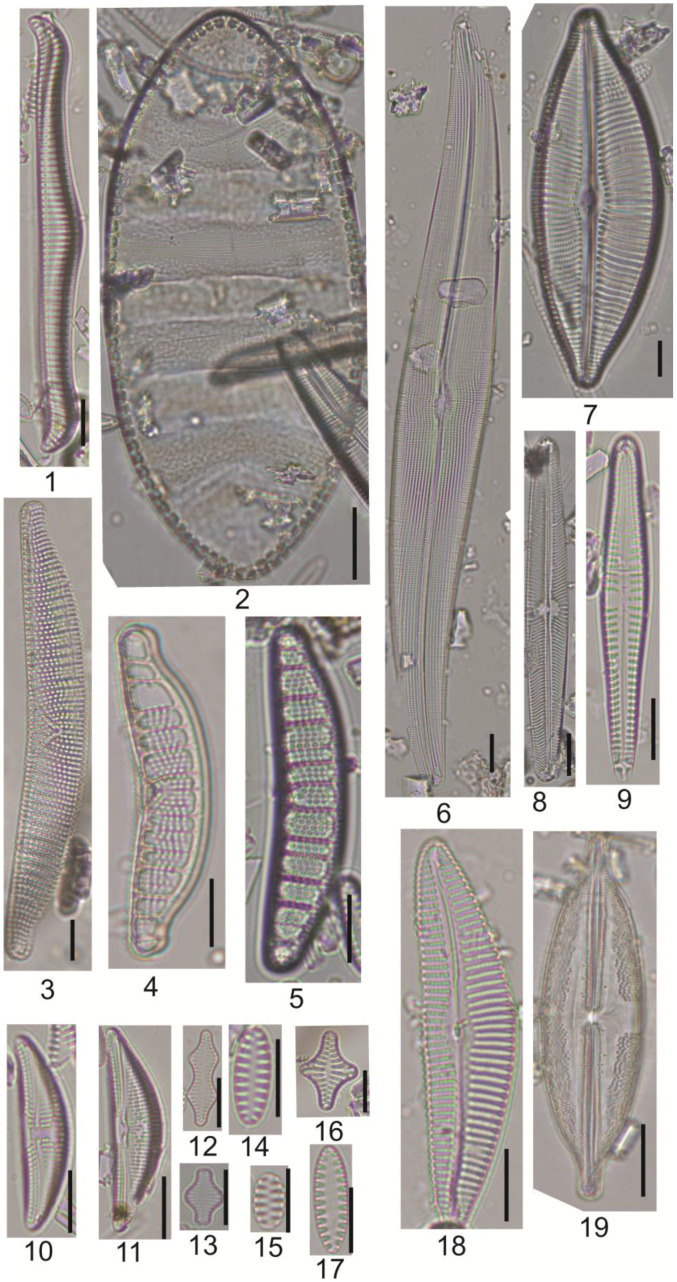
Some diatoms of the Kulikovo section deposits: 1—*Rhopalodia gibba*, 2—*Cymatopleura elliptica*, 3—*Epithemia turgida*, 4—*Epithemia adnata*, 5—*Epithemia frickei*, 6—*Gyrosigma attenuatum*, 7—*Cymbopleura inaequalis*, 8—*Navicula oblonga*, 9—*Gomphonema eileencoxiae*, 10—*Amphora affinis*, 11—*A. copulata*, 12—*Staurosira binodis*, 13—*Staurosira construens*, 14—*Staurosirella ovata*, 15—*Staurosirella martyi*, 16—*Staurosirella leptostauron*, 17—*Staurosirella lapponica*, 18—*Cymbella stigmaphora*, 19—*Anomoeoneis spaerophora*. Bar = 10 µm.

In the sediments of the Kulikovo section at depths of 192–182, 180.5–176 and 29.0–0.0 cm, a small number of diatom valves were found—from 1 to 50 per sample. For the rest of the sedimentary stratum, nine local diatom assemblage zones (LDAZs) were identified, corresponding to changes in the species composition of diatom associations (Figure 2). Images of some of the identified diatoms are shown in Figure 3.

**LDAZ-I** (182–180.5 cm, 13,900–13,870 calBP, one sample). Epiphytic species dominate (84% of all valves), with most of them represented by two species that are indifferent to salinity, including *Pseudostaurosira brevistriata* (64% of all valves), which is indifferent to trophicity, and the eutrophic *Epithemia adnata* (13.5%). The content of benthic species is about 16%. Planktonic species are not represented in this LDAZ. The total content of oligohalobous indifferent species is 89%, that of oligohalobous halophilous species is in the range of tenths of a percent, and that oligohalobous halophobous species is 10%. The total content of eutrophic species is about 14%, mesotrophic—about 1%, oligotrophic—less than 1%, and the content of species indifferent to trophic status is about 84%. The weight concentration is more than 1 × 10^6^ valves/g. In this LDAZ, the Shannon index is 2.5.

**LDAZ-II** (176–169 cm, 13,830–13,750 calBP, two samples). The content of benthic species increases from 40% to 63% up the zone, while the content of epiphytic species decreases from 59% to 33%, and the planktonic species are not represented. The benthic species are dominated by the oligohalobous indifferent, mesotrophic species *Amphora affinis*, widespread in reservoirs of different trophic status (its content decreases from 23 to 9%), and the eutrophic oligohalobous indifferent species *Navicula oblonga* (5–9%). Among the epiphytic diatoms, the most common are the valves of the species *Epithemia adnata* (from 11 to 18% of all valves) and the oligohalobous halophobous mesotrophic species *Cymbopleura inaequalis* (10–13%). The contents of oligohalobous halophobous (from 21 to 36%) and oligohalobous halophilous (from 0 to 13%) species increase up the zone, and the content of oligohalobous indifferent species decreases from 79 to 46%; the content of mesohalobous species increases up the LDAZ-II from 0 to 4%. The contents of mesotrophic (from 9 to 26%), oligotrophic (from 0 to 5%) and eutrophic (from 16 to 22%) species are increasing. The content of species that are indifferent to the trophic conditions of the habitat decreases sharply from 75 to 47%. The weight concentration increases up the zone from 0.05 × 10^6^ to 0.11 × 10^6^ valves/g. Moving down through the zone, the Shannon index decreases from 2.5 to 1.7.

**Subzone IIIa** (169–165 cm, 13,750–13,710 calBP, two samples). Most of the valves belong to benthic species (77–76%), and the content of epiphytic species is 21–22%, while the content of planktonic species is less than 1%. The benthic species are strongly dominated by *Gyrosigma attenuatum* (from 50 to 66% of all valves), a eutrophic oligohalobous indifferent diatom species. The next most abundant species are the benthic eutrophic oligohalobous indifferent *Gyrosigma acuminatum* (7% of all valves in the lower part of the LDAZ) and the epiphytic *Cymbopleura inaequalis* (9–13%). The content of oligohalobous halophobous species ranges from 15 to 16%, and the content of oligohalobous halophilous species decreases upward in the subzone from 13 to 1%, while the content of oligohalobous indifferent species increases to 72–81%. The content of mesohalobous species ranges from the first fractions of a percent to 2%. The content of eutrophic species increases significantly compared to LDAZ-II and ranges from 63 to 74%, while the content of mesotrophic species is 17–19%. Oligotrophic species range from tenths of a percent to 4%, and species living in reservoirs of different trophic status from 14 to 8%. The weight diatom concentration decreases from 0.10 × 10^6^ to 0.05 × 10^6^ valves/g. In this subzone, the Shannon index is 2.1–2.9.

**Subzone IIIb** (165–158.5 cm, 13,710–13,640 calBP, two samples). Compared with LDAZ-IIIa, the content of benthic species valves is lower (it varies from 38 to 56%). In contrast, the content of epiphytic species is higher, although moving higher in the zone, there is a notable decrease in their content from 61 to 42%. The content of planktonic diatoms increases to 0.5%. Among the epiphytic diatoms, valves of oligohalobous species are often found, including oligohalobous indifferent *Staurosirella ovata*, which is indifferent to trophic conditions (its content is from 15 to 32% of all valves), and the eutrophic oligohalobous halophilous species *Melosira varians* (making up 31% of all valves in the lower part of the diatom zone, then decreasing sharply to 1%). Among benthic species, the contents of valves of *Gyrosigma attenuatum* (ranges from 16 to 29%) and *Gyrosigma acuminatum* (15–17%) are high. Moving up the subzone, the content of oligohalobous halophobous species decreases from 11 to 2%, the content of oligohalobous halophilous species decreases from 32 to 2%, the content of oligohalobous indifferent species varies from 66 to 86%, and the content of mesohalobous species barely reaches 1%. The content of eutrophic species is slightly reduced compared to LDAZ-IIIa and amounts to 55–65%. The content of mesotrophic species also decreases and amounts to 6–8%. The content of oligotrophic species ranges from 1 to 4%, while the content of species indifferent to trophic conditions is 21–40%. The weight concentration of the valves increases from 0.012 × 10^6^ to 0.042 × 10^6^ valves/g. Moving up the zone, the Shannon index noticeably decreases from 2.9 to 1.4.

**LDAZ-IV** (158.5–139 cm, 13,640–13,440 calBP, eight samples). Most of the identified diatoms are epiphytic, and their content varies from 56 to 70%. The most numerous valves are *Staurosirella ovata* (from 13 to 44% of the valves), *Pseudostaurosira brevistriata* (the content varies from 3 to 17%), and *Epithemia adnata* (from 2 to 20%). The total content of benthic diatoms ranges from 26 to 43%, Among benthic diatoms, the valves of the *Navicula oblonga* species predominate (from 2 to 13%). The total content of planktonic species does not exceed 1–2%. In this LDAZ, the valves of the oligohalobous indifferent species predominate, their content ranging from 65 to 86%. The content of oligohalobous halophobous species varies from 12 to 32%, while the content of oligohalobous halophilous species does not exceed 2–4%, and the content of mesohalobous species is also 2–4%. The content of eutrophic species is significantly reduced compared to subzone IIIb and ranges from 6 to 18%, while the content of mesotrophic species, in contrast, increases slightly, ranging from 8 to 24%. The content of oligotrophic species is 3–7%, and the content of species indifferent to the trophic status of the habitat varies from 62 to 74%. The weight concentration varies from 1.3 × 10^6^ to 6.4 × 10^6^ valves/g. The Shannon index ranges from 2.0 to 3.0.

**LDAZ-V** (139–126 cm, 13,440–13,310 calBP, five samples). Epiphytic species dominate, with their total content varying from 73% to 90%. Among them, most of the identified valves belong to the species *Staurosirella ovata* (from 26 to 33% of valves) and *Pseudostaurosira brevistriata* (the content varies from 26 to 35%). The content of benthic species varies from 10 to 27%. There is a slight increase in the leaf content of the benthic halophobous mesotrophic species *Amphora copulata* (from 1 to 11%). Planktonic species are not represented in this LDAZ. The oligohalobous indifferent species predominate; their total content ranges from 80 to 93%. The content of oligohalobous halophobous species varies from 3 to 11%, and the content of oligohalobous halophilous species is also variable—it ranges from the tens of a percent to 15%, while the content of mesohalobous species does not exceed 1–3%. The total content of eutrophic diatoms decreases compared to LDAZ-IV and amounts to 3–6%; the content of mesotrophic diatoms varies from 2 to 15%; the content of oligotrophic diatoms does not exceed 1–3%; and the content of diatoms indifferent to the trophic conditions ranges from 80 to 94%. The weight concentration of the valves varies from 5 × 10^6^ to 13 × 10^6^ valves/g. The Shannon index decreases down the zone from 3.2 to 2.1.

**LDAZ-VI** (126–77 cm, 13,310–12,940 calBP, 14 samples). Epiphytic species predominate (their content varies from 57 to 82%), among which, *Pseudostaurosira brevistriata* strongly predominates (from 23 to 46% of all valves). The valves of the species *Staurosirella ovata* (from 4 to 13%) and the oligohalobous halophilous mesotrophic species *Staurosirella lapponica* (from 2 to 18%) are also notable. Among benthic diatoms (from 19 to 42% of all valves), most are represented by the valves of the species *Navicula oblonga* (from 2 to 9%) and *Amphora copulata* (2–4%). The total content of planktonic species does not exceed 1%. The content of oligohalobous halophobous species ranges from 7 to 20%; oligohalobous indifferent species predominate and make up from 61 to 74% of all diatoms; the content of oligohalobous halophilous diatoms varies from 10 to 23%, and the content of mesohalobous species is from 1 to 5%. Diatoms indifferent to trophic conditions predominate, with their content ranging from 71 to 84%. The content of mesotrophic diatoms ranges from 5 to 18%, of oligotrophic—from 1 to 7%, and of eutrophic—from 7 to 17%. The weight concentration of the valves varies widely—from 2–4 × 10^6^ to 18–20 × 10^6^ valves/g, and in the upper part of the zone it decreases to 0.1 × 10^6^ valves/g. The Shannon index ranges from 2.3 to 3.0.

**LDAZ-VII** (77–63 cm, 12,940–12,850 calBP, four samples). The content of benthic diatoms increases up to 46–54%, and most of them are represented by the oligohalobous indifferent species *Amphora ovalis*, which is indifferent to the trophic conditions. Towards the upper part of the LDAZ-VII, the contents of fairly large diatoms valves such as *Cymbopleura inaequalis* (from 2 to 9%), *Gyrosigma attenuatum* (up to 12%) and the oligohalobous halophilous oligotrophic species *Pinnularia flexuosa* (up to 7–14%) also increase. The content of epiphytic species is 45–54%. *Pseudostaurosira brevistriata* prevails among the epiphytic species (its share decreases upward in the zone from 23 to 4%), and the content of valves of the eutrophic species, *Epithemia turgida*, is also high (it increases upward in the zone to 10%). The total content of planktonic species does not exceed 1%. The total content of oligohalobous halophobous species ranges from 16 to 30% of all valves; the content of oligohalobous indifferent species varies from 56 to 73%. The content of oligohalobous halophilous species is 9–12%, and that of mesohalobous species is from 2 to 7%. The content of eutrophic diatoms ranges from 10 to 26%, and the content of mesotrophic diatoms increases slightly up the zone from 15 to 23%. The content of oligotrophic diatoms varies from 4 to 15%, and the content of diatoms indifferent to the trophic conditions varies from 43 to 69%. The weight concentration of the flaps remains relatively low—in the lower part of the LDAZ-VII it reaches 1.5 × 10^6^ valves/g, while in the upper part it decreases to 0.1 × 10^6^ valves/g. The Shannon index rises in the zone from 2.6 to 3.3.

**LDAZ-VIII** (63–41 cm, 12,850–12,720 calBP, seven samples). Almost half of the diatoms or more (from 42 to 80%) are represented by epiphytic species, among which, the valves of the species *Staurosirella ovata* predominate (from 20 to 55%). The content of valves of the oligohalobous halophilous *Staurosira construens*, which is indifferent to the trophic conditions, is also quite high (it decreases from 16 to 3% up the zone). Among benthic species (their content varies from 17 to 45%), the largest proportions of valves belong to the species *Gyrosigma attenuatum* (its content varies within the zone from 2 to 22%), *Amphora affinis* (5–12%), and *A. copulata* (8–10%). In this subzone, the maximum content of planktonic diatoms for the entire section is observed (from 1 to 13%), mainly due to an increase in the number of valves of the mesotrophic species, the indifferent *Aulacoseira islandica*. The content of oligohalobous halophobous species increases upward in the zone from 10 to 20–25%; the content of oligohalobous indifferent species varies from 66 to 85%; the content of oligohalobous halophobous species decreases downward in the LDAZ from 21 to 4%; and the content of mesohalobous species does not exceed 1–4%. The content of eutrophic species varies from 7 to 24%; the content of mesotrophic species increases upward in the zone from 13 to 20–24%; the content of oligotrophic species ranges from 1 to 4%; and the content of species indifferent to the trophic conditions ranges from 59 to 74%. The weight concentration of the valves varies from 0.2 × 10^6^ to 2.0 × 10^6^ valves/g. Moving upward in the zone, the Shannon index declines from 3.2 to 2.2.

**LDAZ IX** (41–29 cm, 12,720–12,650 calBP, three samples). Epiphytic species predominate (from 77 to 95%), most of which are represented by the valves of *Staurosirella ovata* (their content varies from 7 to 56%), and the mesotrophic oligohalobous indifferent species *Staurosirella martyi* (content varies from 9 to 46%). The total content of benthic species varies from 5 to 21%; and the content of planktonic species is 2–4%. The content of oligohalobous halophobous species is 5–7%; the content of oligohalobous indifferent species is 82–85%; the content of oligohalobous halophilous species is 9–11%; and the content of mesohalobous species does not exceed 1%. The content of eutrophic species is in the range 6–8%; the content of mesotrophic species is 24–28%; the content of oligotrophic species does not exceed 1%; and the content of species indifferent to the trophic conditions ranges from 80 to 89%. The weight concentration of the valves varies widely—from 1.4 to 20 × 10^6^ valves/g. Moving upward in the zone, the Shannon index declines from 3.1 to 1.9.

## 4. Discussion

Two parts of the section are distinguished in terms of lithological situation. The lower part (depths 192–162 cm) is less clayey, indicating a more dynamic depositional environment during its formation. Upwards along the section, the proportion of the clay fraction increases, which may indicate calmer sedimentation conditions or an increase in water body depth. A significant increase in the sand fraction at a depth of 78–79 cm probably indicates an environmental event that caused an abrupt short-term change in sedimentation conditions. In general, throughout most of the studied interval, sedimentation conditions were favorable for the development of diatom communities, as well as for the preservation of their valves, except the interval during the Younger Dryas, when dense clay was deposited in the basin. This study showed that, in the Kulikovo section, minimal diatoms are found in the sediments of the Older Dryas and in some layers of the Younger Dryas. In the Allerød sediments, the weight concentration of diatom valves varies widely, from tens of thousands to several millions valves/g. At the beginning of LDAZ-VI, there is a slight decrease in the weight concentration of diatom valves, possibly as a reaction to the cold event GI-1b [39].

The results of studying the Kulikovo section allow us to trace the temporal dynamics of different ecological diatom groups. Epiphytic diatoms, primarily *Pseudostaurosira brevistriata*, *Staurosirella ovata*, *Staurosira construens* and *Staurosirella lapponica*, dominate in diatom complexes almost throughout the entire Allerød. At the same time, such benthic species as *Gyrosigma attenuatum* and *G. acuminatum* predominate in the sediments of the early Allerød in subzones IIIa and IIIb, and the content of primarily *Ampora affinis*, *A. copulata*, *Anomoeoneis sphaerophora*, and *Gyrosigma attenuatum* increases to 20–25% in the Younger Dryas sediments.

Throughout the considered time interval, the predominance of oligohalobous indifferent diatom species is noted. At the same time, in the second half of the Allerød, there was an increase in the content of oligohalobous halophilous species to 10–20%; for example, the content of species such as *Anomoeoneis sphaerophora* and *Staurosira construens* noticeably increases. At the very end of the Allerød and in the Younger Dryas, the content of some oligohalobous halophobous species increased slightly, up to 20–25% in some samples. (Primarily, the content of *Amphora copulata* and *Cymbopleura inaequalis* increased.)

In almost all identified LDAZ, half or more of the diatoms belong to species that are indifferent to the trophic conditions of the habitat. At the same time, the content of the eutrophic species *Gyrosigma attenuatim* increased significantly, up to 30–60% of all diatoms at the beginning of the Allerød, approximately 13,700–13,600 calBP. At the boundary of the Allerød and the Younger Dryas, there was a noticeable increase in the content of mesotrophic (up to 20%) and oligotrophic (up to 15%) diatom species, for example, *Cymbopleura inaequalis*, and *Gomphonema eileencoxiae*.

LDAZ I, III, IIIa, and IIIb, belonging to the first half of the Allerød, are represented by a very small number of samples. Since the LDAZs were isolated using cluster analysis by CONISS, it has a statistical justification. Such fractional clustering may be associated with significant differences in the species composition of diatom complexes due to rapid changes of habitat conditions. Another reason may be the different degree of post-sedimentary preservation of the valves, but this can be true only for subzones IIIa and IIIb, where many diatom valves are represented only by fragments, while in other zones, the valves are well preserved.

In general, the species composition of the diatom assemblages of the Kulikovo section with *Pseudostaurosira brevistriata*, *Staurosirella ovata*, *Epithemia adnata*, *Amphora ovalis*, *Anomoeoneis sphaerophora* etc. throughout the entire Allerød is typical for other synchronous paleoreservoir ecosystems of the region [13,15,16,18]. At the boundary of the Allerød and Younger Dryas, noticeable changes occurred in diatom assemblages: the weight concentration of valves decreased, and the content of epiphytic species decreased, while the content of benthic species increased slightly—up to 15–20%. The beginning of the Younger Dryas is marked by a significant increase in the content of epiphytic species of the Fragilariaceae again. For example, the content of *Staurosirella ovata* increased sharply, reaching its maximum in the section. The identified short-lasting decline of epiphytic species probably testifies to a remarkable water level fluctuation at the start of the Younger Dryas.

The variations in the Shannon index are remarkable. The lowest values are recorded at the beginning of the Allerød: biodiversity decreased significantly in subzone IIIb. This is probably related to a possible increase in the flow rate of the basin and/or water level lowering, as indicated by lithological data, including increases in the content of sand and in the median particle size. These environmental changes led to the prevalence of benthic *Gyrosigma* spp. and eutrophic diatoms, reducing the general species diversity. Starting with LDAZ-IV and continuing throughout the Allerød, the value of the Shannon index varies from 2.0 to 3.3, which indicates a fairly high biodiversity in diatom assemblages. A noticeable but short-lasting (several decades) downward trend in biodiversity at the start of the Younger Dryas is observed, though already since 12,650 calBP, the diversity of species grows as the value of the Shannon index averages 2.5.

In general, it can be said that, as in most Baltic paleoreservoirs with a reconstructed shallow depth [13,15,16,18,19], the role of mesotrophic and eutrophic diatoms in the sediments of the Kulikovo section is notable, in contrast to reservoirs with a greater depth where oligotrophic species predominate [12]. The diatom assemblages of the Kulikovo section, as well as assemblages of other paleoreservoirs in the eastern Baltic [11,12,13,14,15,16,17,18,19], are characterized by the predominance of oligohalobous species. This may indicate low mineralization, not only in moraine lakes, but also in other isolated or semi-isolated basins located along the coast of the BIL.

The pioneer epiphytic species *Staurosirella ovata* and *Pseudostaurosira brevistriata* that predominate in the Kulikovo section are quite common in many sedimentary paleoarchives of the southeastern Baltic, apparently being one of the first diatoms to colonize the bare substrate. Their widespread development also suggests a large amount of dissolved calcium in water, which is confirmed by the results of a comprehensive lithological analysis [21]. In contrast, species of the genus *Gyrosigma* spp., except for in the Kulikovo section, have been observed in large numbers only in the Bølling sediments in Lake Ginkūnai [15], and in the sediments of the second half of the Allerød in the Aleika section [19]. One of the possible explanations for the presence and predominant development of this genus of diatoms may be an increase in the streaming of the reservoirs. Prevailing in Kulikovo in the range of 13,750–13,640 calBP, *Gyrosigma attenuatum* and *G. acuminatum* species are distinguished by their large valve size and ability to live in streaming water with alkaline conditions [40]. The identified diversity of diatoms in the Kulikovo section and further study of their ecological characteristics will provide additional information about the Late Glacial environment and regional paleoclimate of the eastern Baltic.

The significant changes in the species composition of diatom assemblages established for the Kulikovo section in less than a century indicate highly dynamic regional and local natural conditions of the Late Glacial. At the same time, the identified stages are characterized by a very short duration, often comparable to the error of radiocarbon dating. This fact confirms the need for the most detailed investigation of sedimentary archives in order to understand the complexity of the processes that shaped the natural environment at the end of the Pleistocene. With that, when constructing regional reconstructions, it is necessary to take into account the possible asynchrony in the development of biological indicators in reservoirs of different areas, depths and flow rates.

## 5. Conclusions

The essential diversity of Late Glacial diatoms was revealed, and 204 species in total were found as a result of the study of the Kulikovo sediment section in the southeastern Baltic. Among various ecological groups, benthic and epiphytic species belonging to the group of oligohalobous indifferents predominate.

A comparison with available regional data showed that the mass development of pioneer epiphytic diatoms of the Fragilariaceae in the Allerød is common to most of the paleoaquatic ecosystems. A local feature of the Kulikovo paleoreservoir, along with a high diversity of diatoms, is the episode of massive development of *Gyrosigma* spp., benthic diatoms capable of living in streaming water, in the first half of the Allerød.

Study of the Kulikovo section revealed numerous stages with changing species composition of diatom associations in the range of 14,000–12,500 calBP. The relatively short duration of these stages (several decades to a century) indicates a sensitive response of the aquatic ecosystem to rapid changes in the natural environment. The Kulikovo diatom assemblages reacted sensitively not only to the main environmental shifts of the Allerød and Younger Dryas, but probably to smaller-scale climatic events, such as GI-1b and changes in hydrological regimes (water level fluctuations, flow rates) caused by local environmental transformations. The identified diversity of diatoms in the Kulikovo section and further study of their ecological characteristics will provide additional information about the Late Glacial environment and regional paleoclimate of the eastern Baltic.

At the boundary of the Allerød and Younger Dryas, a noticeable change in diatom assemblages occurred: the weight concentration of valves decreased, along with a remarkable short-lasting decline of epiphytic species, probably testifying to a water level fluctuation. It is worth noting that the biodiversity of diatoms remained fairly high, both in the Allerød and the beginning of the Younger Dryas.

## Figures and Tables

**Table 1:** Results of radiocarbon dating of deposits of the Kulikovo section.

Sample	Depth (cm)	Material	Age, ^14^C	Age, calBP
LuS-18436	45	macroremains (wood)	10,940 ± 60	12,773 ± 240
LuS-18462	106	macroremains (wood)	11,060 ± 60	13,102 ± 160
LuS-18461	163	macroremains (wood)	11,790 ± 60	13,693 ± 130
LuS-18460	186	macroremains (wood)	11,980 ± 80	13,957 ± 140
LuS-17811	192	gyttja	12,200 ± 60	14,038 ± 160

**Table 2:** Lithostratigraphy of the Kulikovo section.

Depth (cm)	Layer Description
0–14	Gray dense clay.
14–63	Clay gyttja, from gray to light brown, with rare organic matter. The upper contact is clear and sharp.
63–88	Dark brown peaty gyttja with a thin sandy layer. The upper contact is gradual, clear.
88–158	Clay gyttja, from dark gray to brown, with organic matter and shell. The upper contact is gradual, clear.
158–171	Dark gray clay gyttja with organic matter and shell. The upper contact is gradual.
171–181	Clay dark gray gyttja with rare organic matter and shell. The upper contact is gradual.
181–186	Dark brown peaty gyttja. The upper contact is gradual.
186–192	Clay brown gyttja with rare organic matter. The upper contact is gradual.

## Data Availability

Applicable upon request.

## Abbreviations

The following abbreviations are used in this manuscript:

BIL	Baltic Ice Lake
LDAZ	Local Diatoms Assemblages Zone

## Appendix A

**Table A1:** List of diatom taxa identified in the sediments of the Kulikovo section and the ecology of each taxon. The table was generated according to references [27,34,35,36,40].

№ Taxon		Ecological and Geographical Characteristics of Indicator Organisms						
		Hb	Hl	T	pH	Temp	F	Geo
	Phylum Bacillariophyta Class Bacillariophyceae Subclass Bacillariophycidae Order Achnanthales Family Achnanthidiaceae							
1.	*Achnanthidium eutrophilum* (Lange-Bertalot) Lange-Bertalot 1999	B	ohi	I	alk	E	ind	k
2.	*Crenotia thermalis* (Rabenhorst) Wojtal 2013	B	ohb	M	ind	W	ind	k
3.	*Karayevia clevei* (Grunow) Bukhtiyarova 1999	B	mh	E	alk	–	ind	k
4.	*Karayevia rostrata* (Hustedt) Kulikovskiy & Genkal 2013	B	ohi	E	alk	–	ind	Ha
5.	*Planoplatessa joursacensis* (Héribaud) Kulikovskiy, Glushchenko & Kociolek 2022	B	ohi	M	alk	–	rh	Ha
6.	*Planothidium biporomum* (M.H.Hohn & Hellerman) Lange-Bertalot 1999	B	ohi	I	alk	–	rh	k
7.	*Planothidium frequentissimum* (Lange-Bertalot) Lange-Bertalot 1999	B	ohi	M	alk	–	ind	k
8.	*Planothidium werumianum* Lange-Bertalot & Bąk 2015	B	ohi	E	alk	–	lph	Ha
9.	*Platessa conspicua* (Ant.Mayer) Lange-Bertalot 2004	B	ohi	E	alk	–	lph	k
10.	*Skabitschewskia peragalloi* (Brun & Héribaud) Kuliskovskiy & Lange-Bertalot 2015	B	ohb	I	neu	–	ind	–
Family Bacillariaceae								
11.	*Hantzschia abundans* Lange-Bertalot 1993	B	ohb	M	alk	T	–	k
12.	*Hantzschia amphioxys* (Ehrenberg) Grunow 1880	B	ohi	I	alk	T	ind	k
13.	*Hantzschia calcifuga* E.Reichardt & Lange-Bertalot 2004	B	ohi	I	acd	–	–	Ha
14.	*Nitzschia dissipata* (Kützing) Rabenhorst 1860	B	ohb	E	alk	–	ind	k
15.	*Nitzschia heufleriana* Grunow 1862	B	ohi	E	alk	–	rh	k
16.	*Nitzschia lanceolata* W.Smith 1853	B	mh	E	alk	–	–	–
17.	*Nitzschia linearis* W.Smith 1853	B	ohi	I	alk	T	–	k
18.	*Nitzschia macilenta* W.Gregory ex Greville 1859	B	ohl	I	–	–	–	–
19.	*Nitzschia semirobusta* Lange-Bertalot 1993	B	ohi	I	alk	–	–	k
20.	*Tryblionella angustata* W.Smith 1853	B	ohl	M	alk	–	lph	k
21.	*Tryblionella brunoi* (Lange-Bertalot) Cantonati & Lange-Bertalot 2017	B	mh	I	alk	–	–	Ha
22.	*Tryblionella hungarica* (Grunow) Frenguelli 1942	B	ohi	I	alk	–	ind	k
23.	*Tryblionella scalaris* (Ehrenberg) Siver & P.B.Hamilton 2005	B	ohi	M	neu	–	–	–
Family Cocconeidaceae								
24.	*Cocconeis lineata* Ehrenberg 1849	E	mh	E	alk	–	ind	k
25.	*Cocconeis placentula* Ehrenberg 1838	E	ohi	I	alk	T	ind	k
Order Cymbellales Family Anomoeoneidaceae								
26.	*Anomoeoneis inconcinna* Metzeltin, Lange-Bertalot & Soninkhishig 2009	B	mh	I	–	–	–	–
27.	*Anomoeoneis sphaerophora* Pfitzer 1871	B	ohl	I	acd	W	ind	k
Family Cymbellaceae								
28.	*Cymbella aspera* (Ehrenberg) 1894	E	ohi	E	alk	–	ind	k
29.	*Cymbella helvetica* Kützing 1844	E	ohi	M	alk	–	lph	a, k
30.	*Cymbella lanceolata* C.Agardh	E	ohi	M	alk	–	lph	k
31.	*Cymbella neocistula* Krammer 2002	E	ohb	M	alk	–	–	k
32.	*Cymbella neocistula var. islandica* Krammer 2002	E	ohb	O	neu	–	–	–
33.	*Cymbella neolanceolata* W.Silva 2013	E	ohi	E	alk	–	–	–
34.	*Cymbella parva* (W.Smith) Kirchner 1878	E	ohi	M	ind	–	–	–
35.	*Cymbella perfossilis* Krammer 2002	E	ohb	O	neu	–	–	–
36.	*Cymbella proxima* Reimer 1975	E	ohi	M	–	–	–	k
37.	*Cymbella stigmaphora* Østrup 1910	E	ohb	M	acd	–	–	Ha
38.	*Cymbella subarctica* A.Cleve 1934	E	ohb	O	–	–	–	a-a
39.	*Cymbopleura amphicephala* (Nägeli ex Kützing) Krammer 2003	E	ohb	M	neu	–	ind	b
40.	*Cymbopleura apiculata* Krammer 2003	E	ohi	I	neu	–	lph	Ha
41.	*Cymbopleura designata* (Krammer) Bahls 2019	E	ohb	O	neu	–	–	–
42.	*Cymbopleura inaequalis* (Ehrenberg) Krammer 2003	E	ohb	M	alk	–	–	Ha
43.	*Cymbopleura fluminea* (R.M.Patrick & Freese) Lange-Bertalot & Krammer ex Lange-Bertalot & Genkal 1999	E	ohb	O	neu	–	–	Ha
44.	*Cymbopleura kuelbsii* Krammer 2003	E	ohi	I	–	–	–	–
45.	*Cymbopleura naviculiformis* (Auerswald ex Heiberg) Krammer 2003	E	ohb	O	ind	–	–	b
46.	*Cymbopleura subaequalis* (Grunow) Krammer 2003	E	ohb	I	alk	–	ind	–
47.	*Cymbopleura subcuspidata* (Krammer) Krammer 2003	E	ohi	I	acd	–	–	a-a
48.	*Placoneis amphibola* (Cleve) E.J.Cox 2003	B	ohb	I	ind	C	ind	a-a
49.	*Placoneis clementis* (Grunow) E.J.Cox 1988	B	ohi	E	alk	–	–	b
50.	*Placoneis explanata* (Hustedt) S.Mamaya 2000	B	ohi	M	neu	–	–	k
51.	*Placoneis gastrum* (Ehrenberg) Mereschkowsky 1903	B	ohi	M	ind	–	lph	k
52.	*Placoneis ignorata* (Schimanski) Lange-Bertalot 2000	B	ohi	O	–	–	–	k
53.	*Placoneis paraelginensis* Lange-Bertalot 2000	B	ohi	I	ind	–	–	Ha
54.	*Placoneis parvapolonica* Lange-Bertalot & Wojtal 2014	B	ohi	I	–	–	–	Ha
Family Cymbellales incertae sedis								
55.	*Gomphonella olivaceolacua* (Lange-Bertalot & E.Reichart) R.Jahn & N.Abarca 2019	E	ohi	I	alk	–	ind	Ha
56.	*Gomphonella stauroneiformis* (Grunow) R.Jahn & N.Abarca	E	ohi	I	–	–	–	–
Family Gomphonemataceae								
57.	*Gomphonema acuminatum* Ehrenberg 1832	E	ohi	I	alk	–	ind	k
58.	*Gomphonema brebissonii* Kützing 1846	E	ohi	E	alk	–	lph	Ha
59.	*Gomphonema duplipunctatum* Lange-Bertalot & E.Reichardt 1996	E	ohi	M	acd	–	lph	Ha
60.	*Gomphonema eileencoxiae* (Bahls) Bahls, C.E.Wetzel & Ector 2018	E	ohb	M	neu	–	lph	–
61.	*Gomphonema khentiiense* Metzeltin, Lange-Bertalot & Soninkhishig 2009	E	ohb	O	acd	–	–	–
62.	*Gomphonema lagerheimii* A.Cleve 1895	E	ohb	O	acd	–	–	a-a
63.	*Gomphonema parvulum* (Kützing) Kützing 1849	E	ohi	E	alk	–	–	k
64.	*Gomphonema pseudotenellum* Lange-Bertalot 1985	E	ohi	I	–	–	–	–
65.	*Gomphonema pusillum* (Grunow) Kulikovskiy & Kociolek 2015	E	ohi	I	–	–	–	–
66.	*Gomphonema sarcophagus* W.Gregory 1856	E	ohi	M	neu	–	–	–
67.	*Gomphonema subclavatum* (Grunow) Grunow 1884	E	ohi	I	–	–	–	–
68.	*Gomphonema truncatum* Ehrenberg 1832	E	ohb	E	alk	–	ind	Ha
69	*Gomphonema truncatum var. turgidum* (Ehrenberg) R.M.Patrick 1975	E	ohb	I	–	–	–	k
70.	*Gomphonema utae* Lange-Bertalot & E.Reichardt	E	ohb	I	acd	–	–	Ha
Family Encyonemataceae								
71.	*Encyonema elginense* (Krammer) D.G.Mann 1990	E	ohi	O	acd	–	lph	Ha
72.	*Encyonema lange-bertalotii* Krammer 1997	E	ohb	O	alk	–	–	–
73.	*Encyonema minutum* (Hilse) D.G.Mann 1990	E	ohi	I	neu	–	ind	Ha
74.	*Encyonema obscurum* (Krasske) D.G.Mann 1990	E	ohb	I	–	–	–	–
75	*Encyonema perelginense* Krammer 1997	E	ohb	O	neu	–	–	Ha
76.	*Encyonema silesiacum* (Bleisch) D.G.Mann 1990	E	ohi	I	neu	–	ind	Ha
Family Rhoicospheniaceae								
77.	*Gomphonemopsis exigua* (Kützing) Medlin 1986	E	mh	E	–	–	–	–
Family Witkowskiaceae								
78.	*Witkowskia abiskoensis* (Hustedt) Kulikovskiy, Glushchenko, Mironov & Kociolek 2024	B	ohb	O	ind	–	–	a-a
Order Lyrellales Family Lyrellaceae								
79.	*Petroneis humerosa* (Brébisson ex W.Smith) Stickle & D.G.Mann 1990	B	ohp	I	–	–	–	k
	Order Mastogloiales Family Mastogloiaceae							
80.	*Aneumastus apiculatus* (Østrup) Lange-Bertalot 1999	B	ohb	M	ind	–	–	b
81.	*Aneumastus tusculus* (Ehrenberg) D.G.Mann & Stickle	B	ohb	E	alk	-	lph	k
Order Naviculales Suborder Diploneidineae Family Diploneidaceae								
82.	*Diploneis elliptica* (Kützing) Cleve 1894	B	ohb	O	alk	T	lph	k
Suborder Neidiineae Family Diadesmidaceae								
83.	*Luticola acidoclinata* Lange-Bertalot 1996	B	ohb	O	acd	–	ind	Ha
Suborder Naviculineae Family Naviculaceae								
84.	*Caloneis amphisbaena* (Bory) Cleve 1894	B	ohp	E	alk	–	ind	k
85.	*Caloneis holarctica* Kulikovskiy, Lange-Bertalot & Witkowski 2010	B	ohp	M	–	–	–	–
86.	*Caloneis hyalina* Hustedt 1937	B	ohi	I	neu	–	+	k
87.	*Caloneis molaris* (Grunow) Krammer 1985	B	ohi	I	ind	–	rh	k
88.	*Caloneis silicula var. elliptica* Mayer 1913	B	ohb	O	alk	–	lph	k
89.	*Caloneis tenuis* (W.Gregory) Krammer 1985	B	ohb	O	neu	–	–	Ha
90.	*Caloneis vasileyevae* Lange-Bertalot, Genkal & Vekhov 2004	B	ohb	O	neu	–	–	Ha
91.	*Cavinula variostriata* (Krasske) D.G.Mann & Stickle 1990	B	ohi	I	ind	–	ind	k
92.	*Cavinula vincentii* Antoniades & P.B.Hamilton 2014	B	ohb	O	acd	–	lph	Ha
93.	*Gyrosigma acuminatum* (Kützing) Rabenhorst 1853	B	ohi	E	alk	C	ind	k
94.	*Gyrosigma attenuatum* (Kützing) Rabenhorst 1853	B	ohi	E	alk	–	ind	k
95.	*Hippodonta capitata* (Ehrenberg) Lange-Bertalot, Metzeltin & Witkowski 1996	B	ohi	E	alk	–	ind	–
96.	*Navicula angusta* Grunow 1860	B	ohb	O	acd	–	ind	k
97.	*Navicula associata* Lange-Bertalot 2001	B	ohp	E	alk	–	–	–
98.	*Navicula catalanogermanica* Lange-Bertalot & G.Hofmann 1993	B	ohi	I	–	–	–	–
99.	*Navicula libonensis* Schoeman 1970	B	ohi	E	alk	–	–	–
100.	*Navicula oblonga* (Kützing) Kützing	B	ohi	E	alk	+	ind	k
101.	*Navicula pseudosilicula* Hustedt 1942	B	ohi	I	–	–	–	–
102.	*Navicula radiosa* Kützing 1844	B	ohi	E	neu	T	ind	k
103.	*Navicula reinhardtii* (Grunow) Grunow 1880	B	ohi	I	alk	–	ind	k
104.	*Navicula schweigeri* Bahls 2012	B	ohb	I	neu	–	–	–
105.	*Navicula subalpina* E.Reichardt 1988	B	ohi	M	alk	–	–	–
106.	*Navicula wildii* Lange-Bertalot 1993	B	ohp	O	alk	–	–	Ha
Family Stauroneidaceae								
107.	*Stauroneis acidoclinata* Lange-Bertalot & Werum 2004	B	ohb	O	neu	–	–	–
108.	*Stauroneis acidoclinatopsis* Van de Vijver & Lange-Bertalot 2004	B	mh	I	ind	–	lph	–
109.	*Stauroneis acuta* W.Smith 1853	B	mh	O	alk	–	ind	k
110.	*Stauroneis amphicephala* Kützing 1844	B	ohb	O	ind	–	lph	Ha
111.	*Stauroneis anceps* Ehrenberg 1843	B	ohb	M	neu	–	ind	–
112.	*Stauroneis gracilis* Ehrenberg 1843	B	ohb	M	neu	–	–	Ha
113.	*Stauroneis neohyalina* Lange-Bertalot & Krammer 1996	B	ohb	I	neu	–	lph	–
114.	*Stauroneis phoenicenteron* (Nitzsch) Ehrenberg 1843	B	mh	I	neu	T	ind	k
115.	*Stauroneis producta* Grunow 1880	B	ohi	I	alk	–	lph	Ha
116.	*Stauroneis schulzii* Jousé 1936	B	ohi	–	–	–	–	b
117.	*Stauroneis separanda* Lange-Bertalot & Werum 2004	B	ohi	I	alk	–	ind	–
118.	*Stauroneis sonyae* Kulikovskiy, Lange-Bertalot, Witkowski & Dorofeyuk 2010	B	ohb	I	neu	–	ind	–
119.	*Stauroneis subborealis* Bahls 2010	B	ohb	O	ind	–	lph	–
120.	*Stauroneis supergracilis* Van de Vijver & Lange-Bertalot 2004	B	ohi	E	ind	–	lph	–
Suborder Neidiineae Family Neidiaceae								
121.	*Neidium ampliatum* (Ehrenberg) Krammer 1985	B	ohi	I	neu	–	lph	k
122.	*Neidium bobmarshallense* Bahls 2013	B	ohp	M	neu	–	–	–
123.	*Neidium dubium* (Ehrenberg) Cleve 1894	B	ohb	M	alk	–	–	k
Suborder Sellaphorineae Family Pinnulariaceae								
124.	*Pinnularia divergens* W.Smith 1853	B	ohb	O	neu	–	lph	a-a
125.	*Pinnularia flexuosa* Cleve 1895	B	ohb	O	–	–	–	Ha
126.	*Pinnularia legumen* Ehrenberg 1843	B	ohb	O	neu	–	–	k
127.	*Pinnularia major* (Kützing) Rabenhorst 1853	B	ohi	O	neu	T	ind	k
128.	*Pinnularia marchica* I.Schönfelder 2000	B	ohb	I	–	–	–	–
129.	*Pinnularia neohalophila* Kulikovskiy, Genkal & Mikheeva 2010	B	ohi	E	alk	–	–	Ha
130.	*Pinnularia saprophila* Lange-Bertalot, Kobayasi & Krammer 2000	B	ohi	I	–	–	ind	–
131.	*Pinnularia spitsbergensis* Cleve 1895	B	ohi	O	ind	–	–	a-a
132.	*Pinnularia suchlandtii* Hustedt 1943	B	ohb	O	acd	C	lph	a-a
133.	*Pinnularia subanglica* Krammer 2000	B	ohb	I	acd	–	lph	–
134.	*Pinnularia transversa* (Cleve) Ant.Mayer 1939	B	ohb	O	–	–	–	–
135.	*Pinnularia turfosiphila* E.E.Gaiser & J.R.Johansen 2000	B	ohb	I	acd	–	lph	–
136.	*Pinnularia viridis* (Nitzsch) Ehrenberg 1843	B	ohb	M	neu	T	ind	k
Family Sellaphoraceae								
137.	*Fallacia helensis* (P.Schulz) D.G.Mann 1990	B	ohi	I	alk	–	–	b
138.	*Sellaphora alastos* (M.H.Hohn & Hellerman) Lange-Bertalot & Metzeltin 1996	B	ohi	O	neu	–	–	–
139.	*Sellaphora bacillum* (Ehrenberg) D.G.Mann 2018	B	ohi	M	alk	–	ind	k
140.	*Sellaphora laevissima* (Kützing) D.G.Mann 1989	B	ohi	E	alk	–	ind	–
141.	*Sellaphora pseudopupula* (Krasske) Lange-Bertalot 1996	B	ohi	E	alk	–	–	Ha
142.	*Sellaphora stroemii* (Hustedt) H.Kobayasi 2002	B	ohi	I	alk	E	–	k
Order Rhopalodiales Family Rhopalodiaceae								
143.	*Epithema adnata* (Kützing) Brébisson 1838	E	ohi	I	alk	T	ind	k
144.	*Epithemia argus var. alpestris* (W.Smith) Grunow 1860	E	ohi	M	alk	–	ind	b
145.	*Epithemia frickei* Krammer 1987	E	ohp	E	alk	–	lph	k
146.	*Epithemia hyndmanii* W.Smith 1850	E	ohi	E	alk	–	lph	b
147.	*Epithemia sorex* Kützing 1844	E	ohp	E	alk	T	ind	k
148.	*Epithemia sorex var. gracilis* Hustedt 1922	E	ohp	E	alk	–	ind	b
149.	*Epithemia turgida* (Ehrenberg) Kützing 1844	E	ohi	E	alk	T	ind	k
150.	*Rhopalodia acuminata* Krammer 1987	E	mh	E	alk	–	–	–
151.	*Rhopalodia gibba (Ehrenberg) O.Müller* 1895	E	ohi	I	alk	T	ind	k
Family Stauroneidaceae								
152.	*Craticula ambigua* (Ehrenberg) 1990	B	ohp	E	alk	W	ind	k
153.	*Craticula cuspidata* (Kützing) 1990	B	ohp	M	alk	T	ind	k
Order Surirellales Family Surirellaceae								
154.	*Cymatopleura apiculata* W.Smith	B	mh	E	–	–	+	–
155.	*Iconella bifrons* (Ehrenberg) Ruck & Nakov 2016	B	ohi	I	alk	–	lph	k
156.	*Iconella biseriata* (Brébisson) Ruck & Nakov 2016	B	ohp	I	alk	–	lph	k
157.	*Iconella linearis* (W.Smith) Ruck & Nakov 2016	B	ohi	I	ind	–	–	Ha
158.	*Iconella turgida* (W.Smith) E.Reichardt 2020	B	ohb	I	ind	–	–	b
159.	*Surirella clementis* Grunow 1882	B	mh	I	–	–	–	–
160.	*Surirella hibernica* (W.Smith) Kapustin & Kryvosheia 2019	B	ohi	E	alk	–	–	Ha
161.	*Surirella librile* (Ehrenberg) Ehrenberg 1845	B	mh	I	alk	–	ind	k
162.	*Surirella microlibrile* Van de Vijver, Pottiez & Jüttner 2024	B	mh	I	alk	–	ind	–
163.	*Surirella peisonis* Pantocsek 1902	B	ohi	I	–	–	–	Ha, Pt
164.	*Surirella undulata* (Ehrenberg) Ehrenberg 1845	B	ohi	I	alk	–	ind	k
165.	*Surirella visurgis* Hustedt 1957	B	ohi	M	alk	–	–	–
Order Thalassiophysales Family Catenulaceae								
166.	*Amphora affinis* Kützing 1844	B	ohi	I	alk	T	ind	k
167.	*Amphora copulata* (Kützing) Schoeman & R.E.M.Archibald 1986	B	ohb	I	alk	T	ind	k
168.	*Amphora ovalis* (Kützing) Kützing 1844	B	ohi	I	alk	T	ind	k
169.	*Amphora pediculus* (Kützing) Grunow 1875	B	ohi	I	alk	T	ind	k
Subclass Eunotiophycidae Order Eunotiales Family Eunotiaceae Subclass Eunotiophycidae Order Eunotiales Family Eunotiaceae								
170	*Eunotia minor* (Kützing) Grunow 1881	E	ohi	M	neu	–	ind	Ha
171.	*Eunotia parapraerupta* Lange-Bertalot & Metzeltin 2009	E	ohi	O	ind	–	lph	–
172.	*Eunotia pararepens* Kulikovskiy, Lange-Bertalot, Witkowski & N.I.Dorofeyuk 2010	E	ohb	O	acd	–	lph	–
173.	*Eunotia septentrionalis* Østrup 1897	B	ohb	O	acd	–	lph	a-a
174.	*Eunotia superbidens* Lange-Bertalot 2011	B	ohb	O	acd	–	lph	–
Subclass Fragilariophycidae Order Fragilariales Family Fragilariaceae								
175.	*Fragilaria aequalis* Heiberg 1863	E	ohi	I	alk	–	–	k
176.	*Fragilaria capucina* Desmazières 1830	E	ohi	I	alk	–	ind	k
177.	*Fragilaria crotonensis* Kitton 1869	E	ohi	M	alk	–	lph	k
178.	*Fragilariforma mesolepta* (Rabenhorst) Kharitonov 2005	E	ohi	M	alk	–	–	k
178.	*Fragilariforma virescens* (Ralfs) D.M.Williams & Round 1988	E	ohi	O	neu	–	–	k
179.	*Punctastriata lancettula* (Schumann) P.B.Hamilton & Siver 2008	E	ind	E	alk	–	–	Ha
Order Licmophorales Family Ulnariaceae								
180.	*Ulnaria capitata* (Ehrenberg) Compère 2001	P	ohb	I	alk	–	lph	Ha
181.	*Ulnaria delicatissima* (W.Smith) Aboal & P.C.Silva 2004	P	ohi	I	–	–	ind	k
182.	*Ulnaria ulna* (Nitzsch) Compère	P	ohb	I	alk	–	–	k
Family Staurosiraceae								
183.	*Nanofrustulum trainorii* (E.Morales) E.Morales 2019	E	ohi	I	alk	–	–	–
184.	*Pseudostaurosira brevistriata* (Grunow) D.M.Williams & Round 1988	E	ohi	I	alk	–	lph	k
185.	*Pseudostaurosira elliptica* (Schumann) Edlund, Morales & Spaulding 2006	E	ohi	I	alk	–	ind	–
186.	*Pseudostaurosira parasitica* (W.Smith) E.Morales 2003	E	ohi	I	alk	–	–	k
187.	*Staurosira binodis* (Ehrenberg) Lange-Bertalot 2011	E	ohi	I	alk	–	–	Ha
188.	*Staurosira construens* Ehrenberg 1843	E	ohp	I	alk	T	ind	k
189.	*Staurosira pseudoconstruens* (Marciniak) Lange-Bertalot 2000	E	ohi	I	alk	–	–	–
190.	*Staurosira venter* (Ehrenberg) Cleve & J.D.Möller 1879	E	ohp	E	neu	–	–	k
191.	*Staurosirella lapponica* (Grunow) D.M.Williams & Round 1987	E	ohp	I	alk	–	lph	–
192.	*Staurosirella leptostauron* (Ehrenberg) D.M.Williams & Round 1988	E	ohi	I	alk	–	lph	b
193.	*Staurosirella martyi* (Héribaud) Morales & Manoylov 2006	E	ohi	I	alk	–	ind	k
194.	*Staurosirella ovata* E.A.Morales 2006	E	ohi	I	alk	–	–	Ha
195.	*Staurosirella pinnata* (Ehrenberg) D.M.Williams & Round 1988	E	ohi	I	alk	T	lph	k
Family Staurosiraceae								
196.	*Tabellaria fenestrata* (Lyngbye) Kützing 1844	E	ohb	M	acd	–	ind	k
197.	*Tabellaria flocculosa* (Roth) Kützing 1844	E	ohi	I	acd	E	ind	k
	*Phylum Heterokontophyta* *Subphylum Bacillariophytina* *Class Mediophyceae* *Subclass Thalassiosirophycidae* *Order Stephanodiscales* *Family Stephanodiscaceae*							
198.	*Lindavia affinis* (Grunow) Nakov, Guillory, M.L.Julius, E.C.Theriot & A.J.Alverson 2015	P	ohb	I	neu	–	lph	–
199.	*Lindavia radiosa* (Grunow) De Toni & Forti 1900	P	ohb	M	alk	–	–	k
200.	*Pantocsekiella ocellata* (Pantocsek) K.T.Kiss & Ács 2016	P	ohb	M	alk	–	lph	k
201.	*Pantocsekiella tripartita* (Håkansson) K.T.Kiss & E.Ács 2016	P	ohi	O	ind	C	–	Ha
202.	*Stephanocyclus gamma* (Sovereign) Kulikovskiy, Genkal & Kociolek 2022	P	ohb	M	neu	–	lph	–
203.	*Stephanodiscus alpinus* Hustedt 1942	P	ohi	O	neu	C	lph	–
204.	*Stephanodiscus neoastraea* Håkansson & Hickel 1986	P	ohi	I	ind	–	lph	k

Hb—habitat (B—benthic, E—epiphytic, P—planktonic). Hl—halobian system according to [35,36] (ohb—oligohalobous halophobous, ohi—oligohalobous indifferent, ohl—oligohalobous halophilous, mh—mesohalobous). T—trophic conditions (O—oligotrophic, M—mesotrophic, E—eutrophic, I—indifferent to trophic conditions). pH—pH classification (acd—acidophilous and acidobiontic, neu—circumneitral, alk—alkabiontic and alkaphilous, ind—indifferent to pH). Temp—temperature limitation (W—warm conditions, T—moderate conditions, C—cool conditions, E—everythermal condition species, indifferent to temperature limitations). F—ability to live in flowing water (ind—indifferent to flowability, lph—limnophilous). Geo—preferred geographical zone (Ha—Holarctic, Pt—paleotropic, k—cosmopolitan, b—boreal, a-a—Arctic-Alpine). (–) for all cases means no data.
